# Description of a new species of *Pseudomegischus* van Achterberg from China (Hymenoptera, Stephanidae)

**DOI:** 10.3897/zookeys.601.9499

**Published:** 2016-06-29

**Authors:** Hua-yan Chen, Chun-dan Hong, Cornelis van Achterberg, Zai-fu Xu

**Affiliations:** 1 Department of Entomology, The Ohio State University, 1315 Kinnear Road, Columbus, Ohio 43212, U.S.A.; 2 Nansha Entry-Exit Inspection and Quarantine Bureau, Guangzhou, 511457, P. R. China; 3 Key Laboratory of Resource Biology and Biotechnology in Western China (Northwest University), Ministry of Education; School of Life Sciences, Northwest University, 229 North Taibai Road, Xi’an, Shaanxi 710069, China; 4 Department of Entomology, South China Agricultural University, Guangzhou 510640, P. R. China

**Keywords:** Stephanidae, Pseudomegischus, new species, Oriental Region, China

## Abstract

A new species of the genus *Pseudomegischus* van Achterberg, *Pseudomegischus yunnanensis* **sp. n.**, is described and illustrated from Yunnan Province, China. This is the second species of the genus reported from China. A modified section of the identification key to species of *Pseudomegischus* is added to include the new species.

## Introduction

The genus *Pseudomegischus* van Achterberg, 2002 is a small group of parasitic wasps in the family Stephanidae (Hymenoptera), with only five described species worldwide (Tan et al. 2015). The genus seems to have an Indo-Australian distribution (van Achterberg 2002; Hong et al. 2011; Tan et al. 2015). According to Tan et al. (2015), the potential hosts of the genus include Cerambycidae (Coleoptera) and/or Siricidae (Hymenoptera). Here we report the second species of the genus from the Oriental part of China. We modify the key published by Tan et al. (2015) to include the new species.

## Material and methods

Descriptions of the species have been made under an Olympus SZ61, with lighting achieved through a 27W fluorescent lamp. Photographic images were produced by a digital microscope (VHX-2000c, KEYENCE, Osaka, Japan), and plates were finished with ACDSee 10.0 and Photoshop CS 8.0.1, mostly to adjust the size and background.

Morphological nomenclature follows van Achterberg (2002) and the identification key is modified from the key to species of the genus *Pseudomegischus* in Tan et al. (2015).

Type material is deposited in the Shanghai Entomological Museum, Shanghai, China (SEMC) (Curator: Dr. Hai-sheng Ying).

## Taxonomy

### *Pseudomegischus* van Achterberg, 2002

*Pseudomegischus* van Achterberg, 2002: 169; Aguiar 2004: 73‒74 (list of literature); Hong et al. 2011: 7; Tan et al. 2015: 104. Type species (by original designation): *Stephanus sulcifrons* Schletterer, 1889.

#### Pseudomegischus yunnanensis

Taxon classificationAnimaliaHymenopteraStephanidae

Chen & Xu sp. n.

http://zoobank.org/D24C3295-179F-4732-8A0E-D2D8E0CEB32D

Figs 1‒4 , 5‒11 , 12‒15

##### Material examined.

Holotype, ♀ (SEMC), CHINA: Yunnan, Xishuangbanna, Menglun, 1000 m, 3.VI.2009, No. 34001533.

##### Etymology.

Named after the province of the type locality.

##### Diagnosis.

**Figures 1–4.**
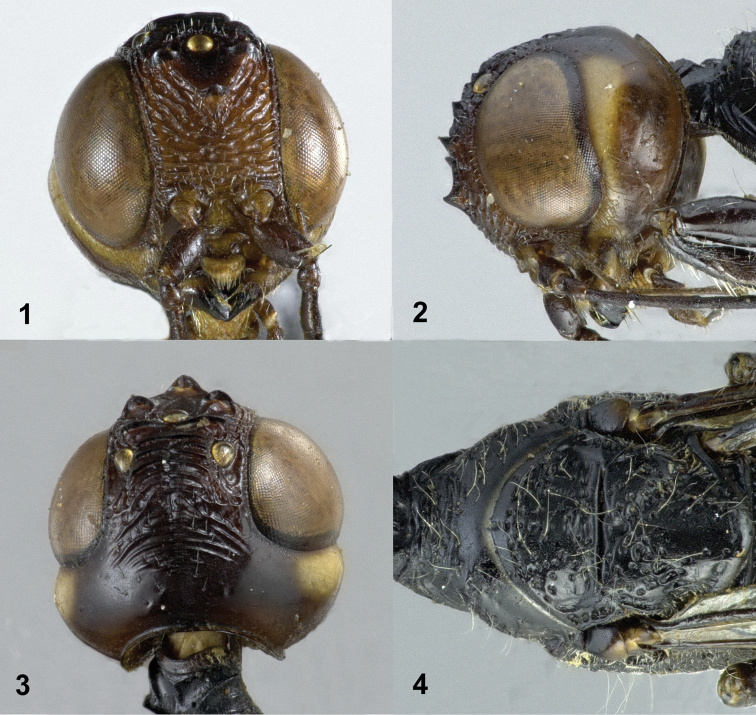
*Pseudomegischus yunnanensis* sp. n., holotype, female. **1** Head frontal **2** head lateral **3** head dorsal **4** pronotum and mesonotum dorsal.

Frons coarsely obliquely rugose near anterior coronal tooth, transversely rugose ventrally (Fig. 1); vertex transversely carinate antero-medially, becoming smooth posteriorly (Fig. 3); middle part of pronotum transversely rugose anteriorly, largely smooth and without distinct carina posteriorly (Fig. 4); scutellum smooth medially, foveolate laterally (Figs 4, 7); propodeum coarsely and densely foveolate, without distinct smooth interspaces (Fig. 7); first tergite largely transversely rugose (Fig. 13).

**Figures 5–11.**
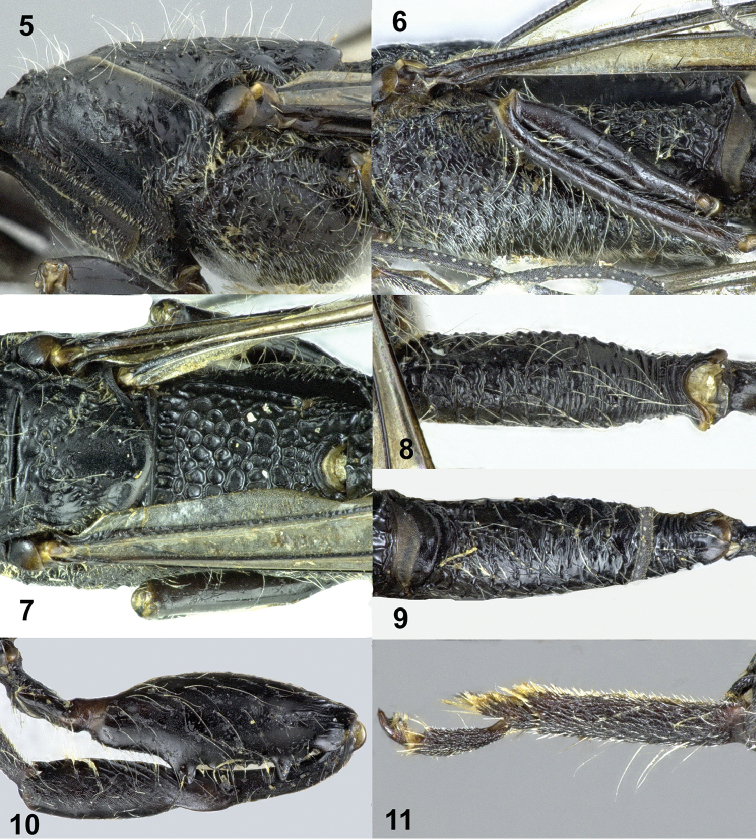
*Pseudomegischus yunnanensis* sp. n., holotype, female. **5** Propleuron and mesopleuron lateral **6** mesopleuron, metapleuron and propodeum lateral **7** scutellum and propodeum dorsal **8** hind coxa dorsal **9** hind coxa lateral **10** hind femur and tibia lateral **11** hind tarsus lateral.

**Figures 12–15.**
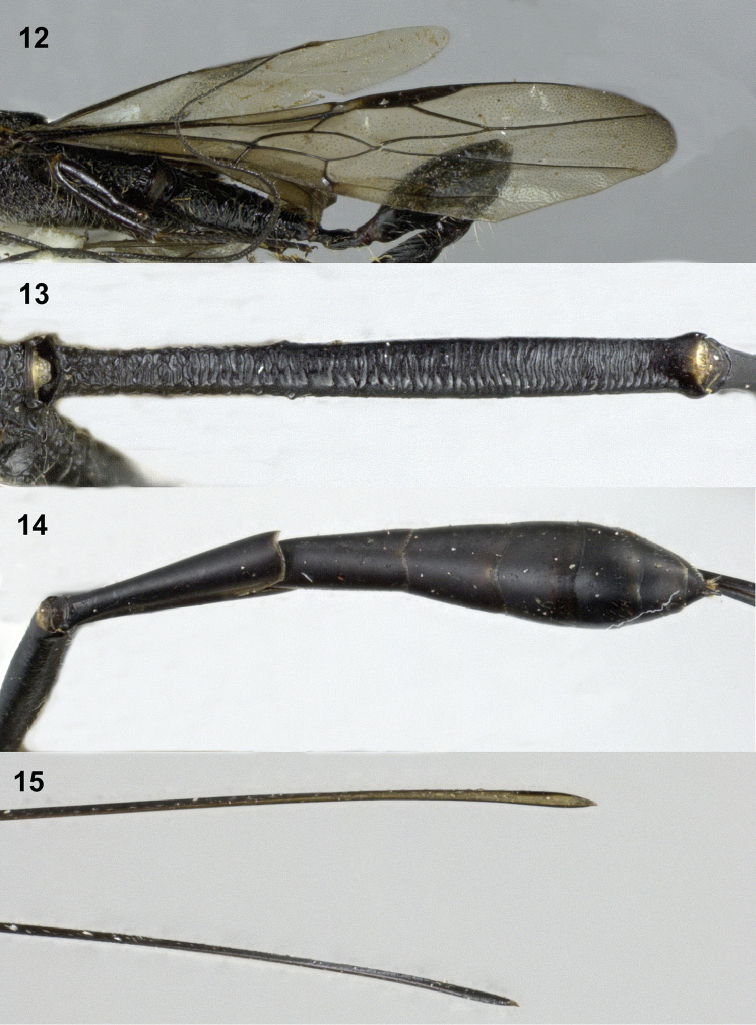
*Pseudomegischus yunnanensis* sp. n., holotype, female. **12** Wings **13** first tergite dorsal **14** metasoma dorsal **15** apex of ovipositor sheaths.

In the key to species of the genus *Pseudomegischus* by Tan et al. (2015), the new species can be included by replacing couplet 3 as follows:

**Table:** 

3	Vein 1-M of fore wing 4.8‒5.4 × as long as vein 1-SR; hind femur with distinct third medium-sized tooth behind large posterior tooth; length of ovipositor sheath about 2.1 × fore wing	***Pseudomegischus sulcifrons* (Schletterer, 1889)**
–	Vein 1-M of fore wing about 3 × as long as vein 1-SR (Fig. 12); hind femur without distinct third medium-sized tooth behind large posterior tooth (Fig. 10); length of ovipositor sheath 2.3‒2.5 × fore wing	**4**
4	Hind basitarsus orange-brown, distinctly contrasting with blackish hind tibia; temple without distinctly differentiated ivory streak; hind femur moderately widened medially in lateral view	***Pseudomegischus celebensis* van Achterberg, 2002**
–	Hind basitarsus largely blackish-brown, not distinctly contrasting with blackish hind tibia (Figs 10, 11); pale or yellow streak of temple well differentiated (Fig. 2); hind femur strongly widened medially in lateral view	**5**
5	Propodeum densely irregularly rugose; first tergite largely smooth except irregular rugae basally and some oblique and fine striae after middle of first tergite	***Pseudomegischus notiochinensis* Tan & van Achterberg, 2015**
–	Propodeum coarsely and densely foveolate, without distinct smooth interspaces (Fig. 7); first tergite largely transversely rugose (Fig. 13)	***Pseudomegischus yunnanensis* sp. n.**

##### Description.

Holotype. *Female*. Body length 21.2 mm; fore wing length 9.6 mm.

*Colour*. Black, except: frons yellowish-brown; temple brown with yellow streak along eye, streak becoming widened near vertex; base of mandible yellow; palpi, vertex, scape, pedicle, tegula and base of femora dark brown; veins and pterostigma largely dark brown, but base of pterostigma ivory; wing membrane slightly brownish; ovipositor sheath blackish apically (Fig. 15).

*Head*. Antenna with 41 segments; frons coarsely obliquely rugose near anterior coronal tooth, transversely rugose ventrally (Fig. 1); three anterior coronal teeth large and acute, both posterior ones arcuate and lamelliform, with two small lobe-shaped carinae on each side in front of both posterior ocelli; vertex transversely carinate antero-medially, becoming smooth posteriorly (Fig. 3); temple non-angulate, smooth and shiny (Fig. 2).

*Mesosoma*. Neck short and robust, transversely rugose, neck at much lower level than middle part of pronotum (Fig. 4); middle part of pronotum transversely rugose anteriorly, largely smooth and without a distinct carina posteriorly; propleuron largely coriaceous with sparse small punctures, shiny and densely setose (Fig. 5); mesonotum sparsely and irregularly foveolate and area between foveae smooth; notauli and median groove distinct; scutellum smooth medially, foveolate laterally; axillae irregularly and rather densely foveolate; mesopleuron distinctly convex, convex part coarsely foveolate-rugose and covered with long whitish setae and dense short setae; metapleuron coarsely foveolate (Fig. 6); propodeum coarsely and densely foveolate, without distinct smooth interspaces (Fig. 7).

*Wings*. Fore wing (Fig. 12): vein 1-M 3.1 × as long as vein 1-SR and curved; vein r ends slightly behind level of apex of pterostigma; first subdiscal cell robust, 3.2 × as long as its maximum width, vein cu-a entirely pigmented.

*Legs*. Hind coxa robust, without tubercle dorsally, transversely and densely rugose (Figs 8, 9); hind femur widened, smooth and with long whitish setae, ventrally with two large acute teeth (the anterior one larger than posterior one) and four denticles in between (Fig. 10); hind tibia 1.2 × as long as hind femur, basal narrow part of hind tibia about 1.2 × as long as widened part, widened part ventrally distinctly obliquely carinate (Fig. 10); hind basitarsus subparallel-sided, length of hind basitarsus 4.8 × as long as wide medially and 3.8 × as long as second tarsal segment (Fig. 11).

*Metasoma*. First tergite 9.0 × as long as its maximum width, 1.6 × as long as second tergite, cylindrical, largely transversely rugose (Fig. 13); remainder of tergites smooth and shiny (Fig. 14); length of ovipositor sheath 2.5 × length of fore wing.

Male. Unknown.

##### Distribution.

Oriental: China (Yunnan).

##### Biology.

Collected in June. Host not known.

## Supplementary Material

XML Treatment for Pseudomegischus yunnanensis
